# Natural history of cow’s milk allergy in Mediterranean area of Turkey

**DOI:** 10.1186/2045-7022-1-S1-P32

**Published:** 2011-08-12

**Authors:** Gulbin Karakoc, Derya Altintas, Mustafa Yilmaz, Seval Kendirli, Dilek Dogruel

**Affiliations:** 1 Cukurova University,Faculty of Medicine, Pediatric Allergy and Immunology, Adana, Turkey

## Background and Objective

Cow's milk allergy (CMA) is the most common food allergy in with most outgrowing by age 3 years. In this study we aimed to define the natural course of CMA and identify the factors that predict outcome.

## Patients and method

Ninety one children with the diagnosis of CMA that were on follow up in Cukurova University, Pediatric Allergy-Immunology Department enrolled to the study. The diagnosis of CMA was made on the basis of a history of symptoms associated with exposure to milk, a positive food challenge, and positive skin prick test and/or specific IgE. Symptoms and clinical findings, cross-reactivity with other proteins, prognosis and risk factors for the persistence were evaluated.

## Results

There were 35 girls and 56 boys with the mean age of 26.4± 19.8 months. Respiratory symptoms were the most common symptoms and seen in 52.7% of the patients, followed by skin symptoms (46.1%). Gastro intestinal symptoms were observed in 10% of the children and anaphylaxis in 3.3%. Cross-reactivity to goat milk, soy milk and beef were 94%, 46% and 76%, respectively. Rates of resolution were 32.3% by age 1 year and 76% by age 3 years. Among the 23 patients with persistent CMA, 20 patients had asthma (86.9%). Inhalant allergen sensitivity developed in 18 of overall patients (19.7%). Coexistence of egg allergy, specific IgE level more than 3.5 kU/L and age onset of the symptoms before than 6 months were determined as significant predictors of outcome.

## Conclusion

In this study, we observed that, 76 % of the children became tolerant to cow's milk by age 3 years. Early age of onset, high specific IgE levels and coexistence of egg allergy were found to be risk factors for the persistence. These children should be followed up for development of asthma and inhalant allergen hypersensitivity.

